# An Introduction to Midwifery

**Published:** 1894-09

**Authors:** 


*An Introduction to Midwifery.* By ARCHIBALD DONALD, M.D. Pp. xii., 188. London: CHARLES GRIFFIN & COMPANY, LIMITED. 1894.

The aim of the author of this book is that of imparting knowledge to students and midwives. This is fulfilled to a certain extent; but we are of opinion that students would have trouble in picking out the information which they may require in their early cases, and that the work is too comprehensive for the training of midwives. Books of the class of Swayne's *Aphorisms* are best for students of both sexes who are beginning their midwifery practice.

Dr. Donald's book is not enough for a medical student reading for examination, but it is a good one for a midwife for reference or for general reading after she has had a little practice. It might in her case take the place of Playfair's work for students. The chapter on antiseptics is excellent, and its directions should be observed by everyone attending midwifery.

There is no doubt that works of this class are useful; but in our opinion it is a mistake to write at one and the same time for students who may become doctors, and for midwives who we trust will always remain such, and who should be trained first of all to do no harm and then to realise as soon as possible when to send for skilled help. As a contribution to this education of midwives, the book may be heartily commended.

